# Ecotopic Expression of the Antimicrobial Peptide DmAMP1W Improves Resistance of Transgenic Wheat to Two Diseases: Sharp Eyespot and Common Root Rot

**DOI:** 10.3390/ijms21020647

**Published:** 2020-01-18

**Authors:** Qiang Su, Ke Wang, Zengyan Zhang

**Affiliations:** The National Key Facility for Crop Gene Resources and Genetic Improvement, Institute of Crop Sciences, Chinese Academy of Agricultural Sciences, Beijing 100081, China; suqiangcaas@163.com (Q.S.); wangke03@caas.cn (K.W.)

**Keywords:** antimicrobial peptides, DmAMP1W, fungal resistance, *Rhizoctonia cerealis*, wheat

## Abstract

Wheat (*Triticum aestivum* L.) is an important staple crop. Sharp eyespot and common root rot are destructive diseases of wheat. Antimicrobial peptides (AMPs) are small peptides with broad-spectrum antimicrobial activity. In this study, we synthesized the *DmAMP1W* gene, encoding *Dahlia merckii* DmAMP1, and investigated the antifungal role of DmAMP1W in vitro and in transgenic wheat. Protein electrophoresis analysis and in vitro inhibition results demonstrated that the synthesized *DmAMP1W* correctly translated to the expected peptide DmAMP1W, and the purified peptide inhibited growths of the fungi *Rhizoctonia cerealis* and *Bipolaris sorokiniana*, the pathogenic causes of wheat sharp eyespot and common root rot. *DmAMP1W* was introduced into a wheat variety Zhoumai18 via *Agrobacterium*-mediated transformation. The molecular characteristics indicated that *DmAMP1W* could be heritable and expressed in five transgenic wheat lines in T_1_–T_2_ generations. Average sharp eyespot infection types of these five *DmAMP1W* transgenic wheat lines in T_1_–T_2_ generations decreased 0.69–1.54 and 0.40–0.82 compared with non-transformed Zhoumai18, respectively. Average common root rot infection types of these transgenic lines and non-transformed Zhoumai18 were 1.23–1.48 and 2.27, respectively. These results indicated that DmAMP1W-expressing transgenic wheat lines displayed enhanced-resistance to both sharp eyespot and common root rot. This study provides new broad-spectrum antifungal resources for wheat breeding.

## 1. Introduction

Bread wheat (*Triticum aestivum* L.) is an important food crop, feeding ≈35% of the world’s population [1]. Sharp eyespot is one of the most serious diseases for wheat production in different regions around the world [2]. Since late 1990s, sharp eyespot has seriously endangered wheat production in China, resulting in 10%–30% yield losses of wheat [3,4]. *Rhizoctonia cerealis*, a necrotrophic fungus, is the major pathogen of sharp eyespot in China. In nature, *R. cerealis* reproduces asexually and exists primarily as vegetative mycelium and/or sclerotia [5]. It can infect the roots and basal stems at any time during the wheat growing season, and in turn can devastate the transport of tissues in stems of wheat and obstruct transportation of nutrition substances [3,6]. Common root rot, caused by the soil-borne fungus *Bipolaris sorokiniana*, is another important disease of wheat [7]. *B. sorokiniana* mainly infects the roots and stem bases of wheat plants. Besides, some *B. sorokiniana* strains also can cause spot blotch, leaf spot disease, seedling blight, head blight and black point in wheat and barley [8,9]. Breeding resistant wheat cultivars are a friendly-environmental approach to protect wheat from fungal diseases. However, it is difficult to breed wheat varieties with resistance to sharp eyespot and common root rot by using traditional method, since no effective resistance accessions are available. Introducing alien genes conferring disease resistance by genetic transformation is an efficient alternative.

To defend against pathogens, plants can produce antimicrobial peptides (AMPs), which have an effect on growth inhibition against microorganisms [10,11,12]. Plant AMPs are structurally small, positively charged and cysteine-rich. AMPs are involved in various antifungal activities in vitro [10,13,14,15]. Some AMPs can directly affect cell membranes of fungi and change their structure, thereby inhibiting growth of the fungi [16,17,18]. For instance, Rs-AFP1, Rs-AFP2 and Rs-AFP3/4, isolated from seeds of *Raphanus sativus*, are the most in-depth-studied antimicrobial peptides in plant disease resistance [19,20,21]. Some studies have shown that Rs-AFP2 can resist to a variety of fungal diseases, such as rice sheath blight, rice blast, wheat sharp eyespot and fusarium head blight [13,20]. Similarly, Br-AMP1 (*Brassica rapa*) [22], Psd1 (*Pisum sativum*) [23], VrD1 (*Vigna radiata*) and MtDef2 (*Medicago trunculata*) [24] are seed specific AMPs, which can inhibit growth of several fungal pathogens. *DmAMP1* was isolated from the seeds of *Dhalia merckii* and was reported to inhibit many fungal pathogens [25,26,27,28]. Bioassay showed that the DmAMP1 peptide extracted from leaves of transgenic papaya inhibited growth of *Phytophthora palmivora* in vitro; thus, ecotopic expression of *DmAMP1* enhanced resistance to this fungal disease in the transgenic papaya [25]. Jha et al. indicated that ecotopic expression of *DmAMP1* in transgenic rice could significantly enhance resistance to blast and rice sheath blight diseases. They demonstrated that *DmAMP1* was expressed independently in the transgenic rice lines and was not associated with rice *PR-1a* gene [26]. With the development of gene synthesis technology, synthetic peptide genes have been more and more used to defend against various fungal and bacterial pathogens [29]. Expression of the synthetic antimicrobial peptide D4E1 improved resistance of transgenic cotton plants to black root rot, because growths of the pathogenic fungi *Fusarium verticillioides* and *Verticillium dahlia* were inhibited by the protein isolated from *D4E1* transgenic plants in vitro [30]. NaD1 (from *Nicotiana alata*) exhibited antifungal activity against *F. vasinfectum* and *V. dahlia* [31]. Ace-AMP1 could effectively enhance resistance against rice blast, sheath blight and bacterial leaf blight in vivo and in vitro, respectively [32]. In addition, Ace-AMP1 could increase resistance to fungal diseases powdery mildew and take-all in transgenic wheat plants [33,34]. However, defense function of DmAMP1 is poorly understood in wheat.

In this report, we aimed to study the inhibition activity of DmAMP1W against wheat disease pathogenic fungi in vitro and in transgenic wheat. The current results indicated that DmAMP1W peptide encoded by the synthesized *DmAMP1W* inhibited against growths of *R. cerealis* and *B. sorokiniana*, and *DmAMP1W*-expressing transgenic wheat plants displayed enhanced resistance to both fungal pathogens.

## 2. Results

### 2.1. Heterogonous Expression and Purification of DmAMP1W

The open-reading-frame sequence (ORF) of *DmAMP1W* was artificially synthesized according to wheat favor codons. It was predicted to encode the DmAMP1 amino acid sequence. The protein sequence analysis showed that the DmAMP1W peptide consists of 84 amino acid (AA) residues, with a molecular weight of 9.26 KD and theoretical isoelectric point (pI) 7.68. SignalP4.0 and NCBI blastp showed that the DmAMP1W protein contained a signal peptide (locating number 1–28 AA residues) and a Knot1 domain (at 30 to 78 AA) harboring eight cysteines (Supplemental Figure S1). The DmAMP1W mature peptide was identical to that of DmAMP1 [35] (Supplementary Figure S2).

**Figure 1:**
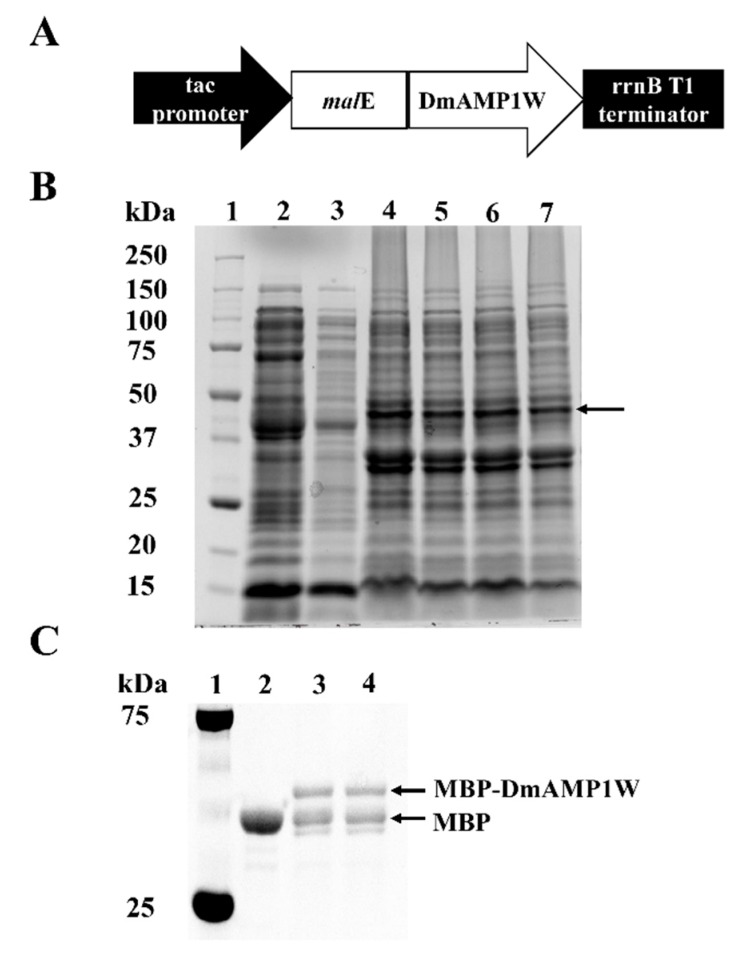
Expression and purification of MBP-DmAMP1W. (**A**) Construction of pMAL-C5X-DmAMP1W prokaryotic expression vector. In *Escherichia coli*, *mal*E-*DmAMP1W* was predicted to encode the recombinant protein MBP-DmAMP1W. (**B**) SDS-PAGE (12%) analysis of MBP and MBP-DmAMP1W fusion protein. Lane 1, protein marker. Lane 2 and 3, the supernatant of cell lysate of pMAL-C5X DE3. Lanes 4–7, the supernatant of cell lysate of pMAL-C5X-DmAMP1W DE3. The black arrow represents the target protein. (**C**) SDS-PAGE analysis of the purified MBP and MBP-DmAMP1W fusion protein. Lane 1, protein marker. Lane 2, purified MBP tag protein. Lane 3 and 4, purified MBP-DmAMP1W fusion protein.

The ORF of *DmAMP1W* was sub-cloned and fused with the MBP tag in the prokaryotic expression vector pMAL-C5X (Figure 1A). The *mal*E-*DmAMP1W*, where *mal*E encodes the MBP tag, was predicted to encode the recombinant protein MBP-DmAMP1W. In order to obtain more amounts of MBP-DmAMP1W soluble fusion protein, we optimized the expression conditions. We established the optimum cultivation and induction conditions, when the culture cell density was OD600 = 0.6, the culture was induced by the addition of 0.1 mM isopropyl-β-D-thiogalactopyranoside (IPTG) under 28 °C and for ≈ 12 h at 180 rpm. Sodium dodecyl sulphate-polyacrylamide gel electrophoresis (SDS-PAGE, 12%) analysis showed that a clear expression protein band was found and should be MBP-DmAMP1W (Figure 1B). The MBP and MBP-DmAMP1W proteins were purified using Amylose Resin and were eluted by the MBP elution buffer, respectively. When these two solubilized proteins were monitored by gel electrophoresis, the sizes of the proteins were consistent with theoretical molecular masses of MBP and MBP-DmAMP1W (Figure 1C). MBP and MBP-DmAMP1W proteins were dissolved in MBP elution buffer (0.04% maltose solution) to final concentration of 65 μg/mL.

### 2.2. DmAMP1W In Vitro Inhibits Growth of R. cerealis and B. sorokiniana

**Figure 2:**
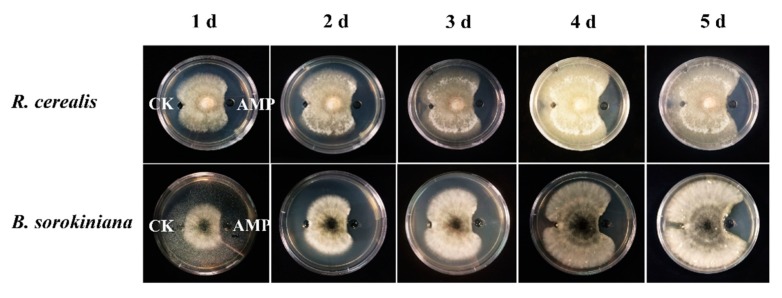
Inhibitory activity of DmAMP1W against *Rhizoctonia cerealis* and *Bipolaris sorokiniana.* The growth of pathogenic fungi mycelium within five days after inoculation was exhibited from left to right. The fungal mycelia on plates were treated by 65 μg/mL MBP and 65 μg/mL MBP-DmAMP1W, respectively. CK and AMP on the mediums represent MBP and MBP-DmAMP1W treatments, respectively. These assays were conducted three times with similar results.

To examine inhibiting activity of the DmAMP1W against growths of fungal pathogens of wheat sharp eyespot and common root rot, 65 μg/mL MBP and 65 μg/mL MBP-DmAMP1W were injected into the small pores of PDA mediums, respectively, and then *R. cerealis* and *B. sorokiniana* were inoculated onto the PDA mediums. All treatments were performed three times. The results indicated that mycelium growths of *R. cerealis* and *B. sorokiniana* in PDA mediums injected with MBP-DmAMP1W were obviously inhibited compared with the MBP-treated (control) parts (Figure 2). Interestingly, three days after inoculation, when the mycelium filled up MBP-treated PDA medium, the areas treated with MBP-DmAMP1W formed inhibition zones, and they lasted for 10 d.

### 2.3. Generation and Molecular Characterization of DmAMP1W Transgenic Wheat

**Figure 3:**
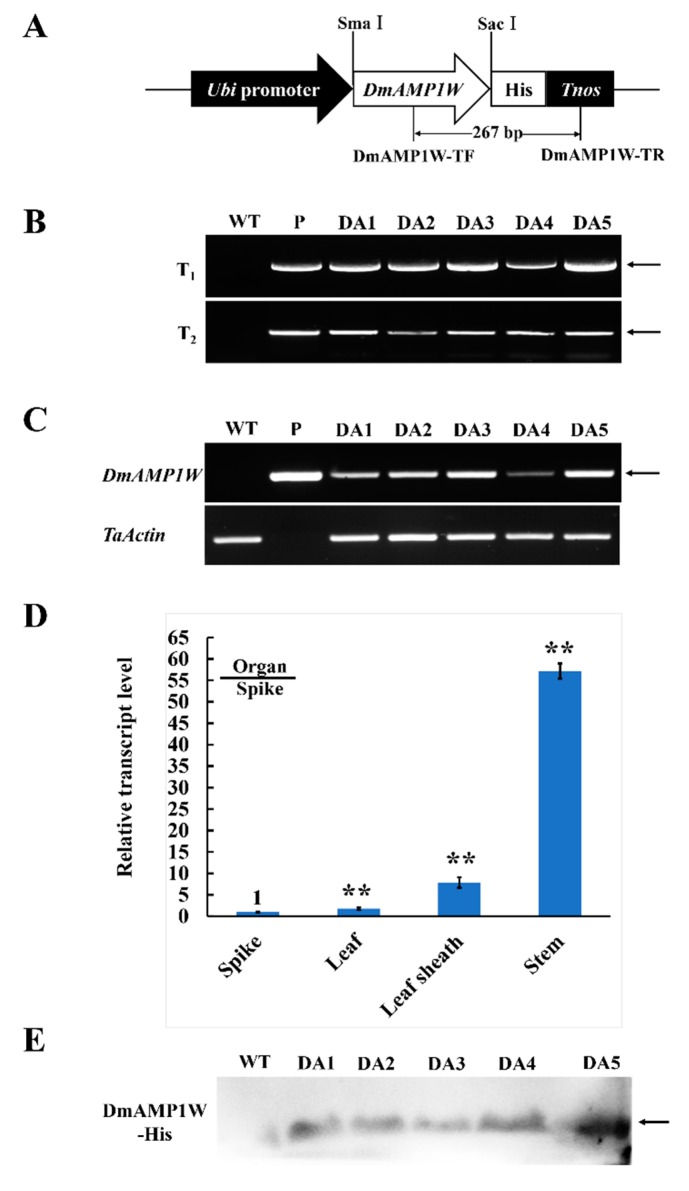
Plant expression vector and molecular characterization in *DmAMP1W* transgenic wheat plants. (**A**) Plant expression vector pWMB122:DmAMP1W–His. *Tnos*, 3′ untranslated terminator region of the *Agrobacterium* tumefaciens nopaline synthase gene. The arrow indicates the fragment amplified in the PCR detection. (**B**) PCR patterns of *DmAMP1W* in T_1_–T_2_ transgenic plants. WT indicates the non-transformed Zhoumai18. P represents pWMB122:DmAMP1W–His vector as positive control. DA1, DA2, DA3, DA4 and DA5 represent *DmAMP1W* transgenic wheat lines. (**C**) Semi-quantitative RT-PCR assays of *DmAMP1W* transcript in transgenic wheat. The amplification of semi-quantitative RT-PCR took 27 cycles. WT indicates the non-transformed Zhoumai18. P represents pWMB122:DmAMP1W–His vector as positive control. DA1, DA2, DA3, DA4 and DA5 represent *DmAMP1W* transgenic wheat lines. (**D**) qRT-PCR analysis of the organ expression pattern of *DmAMP1W* in transgenic wheat lines at 30 d post *R. cerealis* inoculation. Total RNA was extracted from spike, leaf, leaf sheath and stem organs of the five transgenic wheat lines. Each sample of three technique replications was averaged and statistically treated (Student’s *t*-test; ** *p* < 0.01). Error bars represents standard errors. (**E**) DmAMP1W–His fusion protein in transgenic plants in T_2_ and recipient Zhoumai18 was monitored by immunoblot analysis with the polyclonal anti-His antibody.

The monocot expression vector pWMB122-*DmAMP1W* (Figure 3A), encoding DmAMP1W–His fusion protein, was used to transform a high-yield wheat cv. Zhouami18 via *Agrobacterium*-mediated method. As expected, transgenic wheat plants were generated. Using PCR by the primers specific to the transformation construct, an amplified product with 267-bp length was present in all the positive plants and the positive control vector (pWMB122-*DmAMP1W*), but absent in the negative plants and non-transformed (WT) recipient Zhouami18 (Figure 3B). Simi-quantitative RT-PCR results indicated that the fragment specific to the *DmAMP1W* transcript was observed in both five positive transgenic wheat lines and pWMB122-*DmAMP1W* plasmid, but absent in WT Zhouami18. Further RT-qPCR analysis indicated that *DmAMP1W* could be expressed in all the tested organs (spike, leaf, leaf sheath and stem) of the transgenic wheat plants, and the highest expression occurred in the stems (Figure 3D). Western blot (immunoblot) analysis exhibited that DmAMP1W–His fusion protein was expressed in these five transgenic wheat lines but not in WT Zhoumai18 (Figure 3E).

### 2.4. DmAMP1W Expression Improves Resistance of Transgenic Wheat to Sharp Eyespot and Common Root Rot

**Figure 4:**
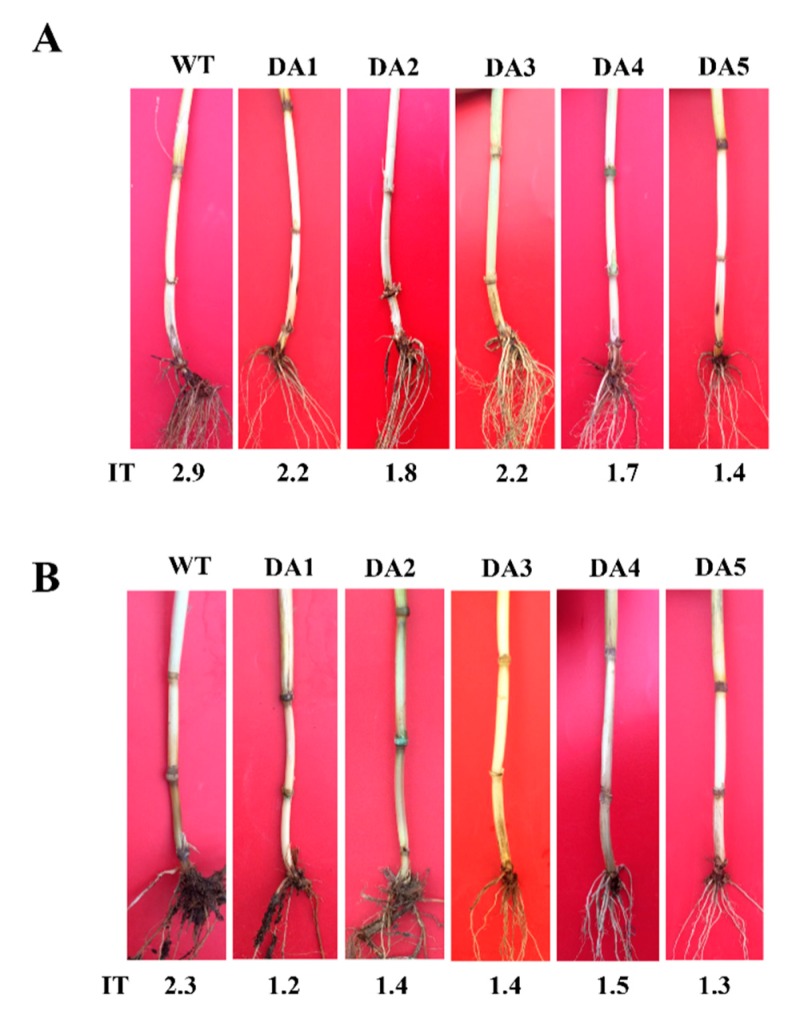
Typical symptoms of sharp eyespot and common root rot in *DmAMP1W* transgenic and non-transformed wheat plants. (**A**) Typical symptoms of sharp eyespot in *DmAMP1W* transgenic wheat lines and non-transformed wheat cv. Zhoumai18. Zhoumai18 was the transgenic recipient. DA1, DA2, DA3, DA4 and DA5 represent *DmAMP1W* transgenic wheat lines. IT indicates infection type. WT represents the recipient Zhoumai18. (**B**) Typical symptoms of common root rot in *DmAMP1W* transgenic wheat lines and non-transformed wheat cv. Zhoumai18. DA1, DA2, DA3, DA4 and DA5 indicate *DmAMP1W* transgenic wheat lines. IT represents infection type. WT represents the recipient Zhoumai18.

To investigate defense ability of *DmAMP1W* in wheat against *R. cerealis*, we assessed resistance of *DmAMP1W* transgenic wheat plants in T_1_–T_2_ generations to sharp eyespot after the pathogen inoculation. The assessment results indicated that these five *DmAMP1W* transgenic wheat lines displayed significantly enhanced-resistance to *R. cerealis* compared to WT Zhoumai18. According to the assessment results of T_1_ resistance to sharp eyespot, five lines with heightened-resistance, namely DA1, DA2, DA3, DA4 and DA5, were selected to further assess in T_2_ generation. Average infection types (Its) of these five transgenic wheat lines (DA1, DA2, DA3, DA4 and DA5) in T_1_–T_2_ generations were 1.37–2.22 and 2.30–2.72, while those of WT Zhoumai18 plants were 2.91 and 3.12. The average ITs of these five transgenic wheat lines decreased 0.69–1.54 and 0.40–0.82 compared with WT Zhoumai18, respectively (Table 1). Meanwhile, the disease severities and indices of these *DmAMP1W* transgenic wheat plants were significantly less and decreased than those of WT Zhoumai18 plants (Figure 4A, Table 1). The above results proved that expression of *DmAMP1W* significantly increased resistance of the transgenic wheat plants to sharp eyespot.

Moreover, the common root rot severity assessment displayed that, compared with WT Zhoumai18, these five transgenic lines (DA1, DA2, DA3, DA4 and DA5) in T_2_ generation exhibited significantly enhanced resistance to *B. sorokiniana* infection (Figure 4B); the average ITs and common root rot disease indices of these five transgenic wheat lines were 1.23–1.48 and 24.60–29.60, whereas the average IT and disease index of WT Zhoumai18 were 2.27 and 45.40, respectively (Table 2). These results suggested that expression of *DmAMP1W* improved resistance of the transgenic wheat plants to common root rot. 

## 3. Discussion

Wheat is one of the most important staple crops globally. Wheat often is inflicted by many abiotic and biotic stresses. The pathogenic fungi *R. cerealis* and *B. sorokiniana* seriously reduce wheat yield in many regions of the world. It is difficult to breed wheat varieties with resistance to both diseases because of the lack of high resistance genes in wheat. It is an effective way to introduce genes with good performance to solve this problem. Broad-spectrum and stable resistance make AMPs candidates for transgenic breeding [21,36]. Our earlier works demonstrated that transgenic wheat plants expressing exogenous AMP genes exhibited increased resistance to fungal pathogens, such as *Gaeumannomyces graminis*var. *tritici* [37], *Fusarium graminearum* and *R. cerealis* [20]. Therefore, generation of transgenic crops expressing AMPs has been an effective approach against fungal diseases [38,39,40,41]. Although Jha et al. reported that transgenic rice lines expressing *DmAMP1* gene improved resistance to the diseases rice blast and rice sheath blight [26], it has not been verified that the resistance abilities of DmAMP1 to fungi diseases in wheat are real.

The correct processing, stable and efficient expression of antimicrobial peptides in transgenic plants are of great significance for the antifungal activity in plants [26]. In this study, according to wheat preference codons, we synthesized *DmAMP1W* gene sequence that encodes DmAMP1 mature peptide, and a signal peptide sequence was added at the N terminal in order to secretion of DmAMP1W. We chose DmAMP1 because of its clear disease resistance mechanism and multiple verifications that it can effectively improve plant resistance to fungal diseases [25,26,27,28]. Heterologous expression and SDS-PAGE analyses indicated that the MBP-DmAMP1W recombinant protein was highly expressed in *Escherichia coli*. In vitro testing demonstrated that the synthesized *DmAMP1W* had the expected activity and DmAMP1W inhibited effectively against mycelium growth of *R. cerealis* and *B. sorokiniana*. Interestingly, DmAMP1W could inhibit the fungal growth in vitro for ten days. In the transformed vector pWMB122-*DmAMP1W*, *DmAMP1W* was controlled by the maize ubiquitin promoter. Through *Agrobacterium*-mediated transformation, we generated *DmAMP1W* transgenic wheat lines and investigated the antifungal ability in transgenic wheat lines. *DmAMP1W* transgenic wheat lines exhibited enhanced-resistance to *R. cerealis* and *B. sorokiniana* compared to WT Zhoumai18, which supported our experiments in vitro. Expression of *DmAMP1* in transgenic rice and papaya boosted resistance against the fungal pathogens *M. oryzae*, *B. sorokiniana* [26], and *P. palmivora* [25], respectively. Our results broaden the antifungal spectrum of DmAMP1 and support the conclusion of previous studies [25,26]. These reports proved that *DmAMP1* could be used to improve resistance to fungal diseases in various transgenic plant species.

Given the great harm and cost of plant protection chemicals, researchers are more inclined to develop efficient and eco-friendly methods to control plant pathogens [26]. Generation of a broad-spectrum of disease resistant crops has always been a goal for breeders. In previous studies, Rogozhin et al. found that Ns-D2 inhibited hyphal growth of *B. sorokiniana*, *F. oxysporum*, *B. cinerea* and *Ph. Infestans*, and its antifungal activity is similar to Rs-AFP1 and Rs-AFP2, two radish AMPs [42]. *Rs-AFP2* and *DmAMP1* were widely transformed into several crops, such as wheat [20], rice [13,26], maize [43] and papaya [25], and these transgenic crop plants exhibited enhanced resistance to various pathogens [13,20,25,26,43]. In this study, *DmAMP1W* transgenic wheat lines in T_1_–T_2_ generations were monitored by PCR, semi-quantitative RT-PCR and western blot assays. The results demonstrated that *DmAMP1W* could be expressed in five *DmAMP1W* transgenic wheat lines in T_1_–T_2_ generations. Accordingly, disease assessment results in two consecutive years exhibited that these five *DmAMP1W* transgenic wheat lines, DA1, DA2, DA3, DA4 and DA5, displayed enhanced resistance to sharp eyespot in T_1_–T_2_ generations. In detail, sharp eyespot infection types in five transgenic lines decreased 0.69–1.54 and 0.40–0.82 compared with WT Zhoumai18, respectively. Also, expression of *DmAMP1W* could improve resistance of transgenic wheat to common root rot, whose common root rot infection types decreased 0.79–1.04 compared with WT Zhoumai18. The expression assay demonstrated that *DmAMP1W* transcript at the adult-plant stage was widespread in the tested organs (spike, leaf, leaf sheath and stem) of the transgenic wheat and the highest expression might be in the stems. In view of *R. cerealis* and *B. sorokiniana* pathogens mainly attack the base stems and roots of wheat plants, and the high expression of *DmAMP1W* in stems is conducive to defense of transgenic wheat against infection of these pathogens. Although we tried to examine the expression of *DmAMP1W* in all the organs of the transgenic wheat at the adult-plant stage, unfortunately, it was difficult to extract RNA from the root for analysis in this stage. Therefore, to clarify the issue further, we need to examine *DmAMP1W* expression in organs of the transgenic wheat plants at the seedling stage in the future. Taken together, the results proved that DmAMP1W peptide has resistance activity to both *R. cerealis* and *B. sorokiniana* in vitro and in transgenic wheat.

In conclusion, we synthesized the *DmAMP1W* gene according to wheat preference codons, and characterized its antifungal role in vitro and in transgenic wheat. Our results demonstrated that the synthesized *DmAMP1W* could correctly translate to the expected peptide. Expressing DmAMP1W peptide could inhibit mycelia growth of *R. cerealis* and *B. sorokiniana* in vitro. Moreover, we probed into the defensive function of *DmAMP1W* in transgenic wheat against these fungal pathogen challenges, and the results demonstrated that ectopic expression of *DmAMP1W* increased resistance of the transgenic wheat to sharp eyespot and common root rot. In addition, *DmAMP1W* expression was the highest in the stems of the transgenic wheat plants, which benefited to control of the both soil-borne diseases. Our work provides new broad-spectrum antifungal accessions for wheat breeding against sharp eyespot and common root rot.

## 4. Materials and Methods

### 4.1. Plant Materials and Pathogenic Fungi

A highly-yield wheat cultivar Zhoumai 18 was used as the transformed recipient in this research. The common root rot pathogenic fungus *Bipolaris sorokiniana* strain ACC30209 was preserved in our laboratory. The sharp eyespot pathogenic fungus *Rhizoctonia cerealis* strain RC0301 was provided by Huaigu Chen and Shibin Cai in Jiangsu Academy of Agricultural Sciences, China. 

### 4.2. Construction of pMAL-C5X-DmAMP1W Prokaryotic Expression Vector

The ORF sequence of *DmAMP1W* gene was amplified by PCR using the pMBW122-*DmAMP1W* plasmid as the template and with the primers (DmAMP1W-BamHI-F-: 5′-CTAGGATCCATGGTGAACAGGTCCGT-3′, DmAMP1W-EcoRI-R: 5′-CACGAATTCTCAG CAGTTGAAGTAGCAGA-3′, and the underlined stands for restriction sites to introduce *BamH*I and *EcoR*I restriction sites). Subsequently, it was subcloned into the *BamH*I and *EcoR*I sites of pMAL-C5X vector and was fused with MBP epitope tag. The recombinant vector was confirmed by sequencing and named pMAL-C5X-DmAMP1W.

### 4.3. Induction, Extraction and Purification of MBP-DmAMP1W Fusion Proteins

The pMAL-C5X-DmAMP1W and pMAL-C5X were transformed in *E. coli* DE3 competent cells. The transformed competent cells were grown in 200 mL LB medium (100 μg/mL ampicillin) until OD600 was 0.6, and then transformed competent cells were induced with 0.1 mM concentrations of IPTG under 28 °C for 12 h at 180 rpm.

The culture was divided into 50 mL tubes and then centrifuged at 4000 × *g* for 20 min to harvest cells. The cells were suspended in PBS buffer (pH 7.4) and divided in 1.5 mL tubes with 10 μL lysozyme (Thermo Fisher Scientific, Boston, MA, USA). Then, the cells were broken up by freeze-thaw method. The cell debris was centrifuged for 15 min at 4 °C and 12,000 × rpm. The clear supernatant, containing soluble fraction, was collected and purified; 100 μL Amylose Resin (NEB, Ipswich, MA, USA) was flowed with PBS buffer (pH 7.4) 3 times, and then the supernatant solution was incubated with Amylose Resin at 4 °C with end-over-end rotation overnight. The MBP and MBP-DmAMP1W were eluted by the MBP elution buffer (0.04% maltose solution), respectively. BCA method was used to confirm the protein concentration referring to the Easy II Protein Quantitative Kit (TransGen Biotech, Beijing, China) instruction. After that, a total of 10 μL of the purified proteins were analyzed by 12% SDS–PAGE (Bio-Rad, Hercules, CA, USA).

### 4.4. MBP-DmAMP1W Antifungal Activity Assay In Vitro

The mycelial growth inhibition method [44] was used to indicate the antifungal activity of the sample with the radius of the inhibition zone. The *R. cerealis* and *B. sorokiniana* were inoculated in the center of PDA medium to culture for 2 d at 25 °C. After that, two symmetrical holes were punched in the medium, and MBP and MBP-DmAMP1W were injected into the hole, respectively. After the proteins infiltrated the medium, the mycelial growth was observed for 10 days.

### 4.5. Generation of DmAMP1W Transgenic Wheat Plants

*DmAMP1W* was artificially synthesized according to the amino acid sequence of an antimicrobial peptide *DmAMP1* isolated from dahlia. The *DmAMP1W*, with a *His* epitope tag, was subcloned into the *Sam* I and *Sac* I sites of *Agrobacterium*-mediated transformation vector pWMB122 [45]. In the resulting transformed vector pMBW122-*DmAMP1W*, the *DmAMP1W–His* fusion gene was driven by the maize ubiquitin (Ubi) promoter and terminated by *Agrobacterium* tumefaciens nopaline synthase gene (*Tnos*).

### 4.6. DNA and RNA Extractions and cDNA Synthesis of Wheat

For molecular analysis of transgenic wheat plants, the leaves of *DmAMP1W* transgenic wheat plants were collected at jointing stage to isolate DNA and RNA. Genomic DNA was extracted from the leaves using the CTAB method [46]. To analyze the tissue expression pattern of *DmAMP1W* in transgenic wheat lines, the transgenic wheat plants at the tillering stage were inoculated with *R. cerealis* RC0301. At 30 dpi, the roots, stem, leave, and spike were collected and deployed to RNA extraction. Total RNA was extracted using TRIzol (Invitrogen, Burlington, ON, Canada). The RNA samples were reverse-transcribed to cDNA using FastQuant RT Kit (Tiangen, Beijing, China) according to the manufacturer’s instructions.

### 4.7. PCR and RT-(q)PCR Analyses of DmAMP1W Transgene in Wheat

The *DmAMP1W* transgenic plants was monitored by PCR using transgene-specific primers (DmAMP1W-TF: 5′-ATGAAGTTGCCGGGATTGC-3′ located in the *DmAMP1W* coding region; DmAMP1W-TR: 5′-AAAACCCATCTCATAAATAACG-3′ located in *Tnos*). The PCR reaction was set as follows: 94 °C for 3 min; 94 °C for 30 s, 54 °C for 30 s, and 72 °C for 2 min, for 32 cycles; and a final extension at 72 °C for 5 min.

Semi-quantitative RT-PCR was used to analyze the transcription of *DmAMP1W* in different transgenic lines. *TaActin* (TaActin-F: 5′-CACTGGAATGGTCAAGGCTG-3′ and TaActin-R: 5′-CTCCATGTCATCCCAGTTG-3′) was used as internal control. In the 25 μL amplification reaction, the amounts of templates added were adjusted (Zhoumai18, 1.0 μL; DA1, 0.75 μL; DA2, 2.0 μL; DA3, 5.0 μL; DA4 7.0 μL; DA5, 1.5 μL) according to the *TaActin* electrophoretic brightness in different lines. And the following materials were added: 12.5 μL of 2 × PCR Mixture; 0.75 μL of forward and reverse primers (gene-specific primers for *DmAMP1W* were: DmAMP1W-RT-57F: 5′-TGTGCTTGCCGTTC CTCA-3′ and DmAMP1W-RT-211R: 5′-CATCTCCGACATCGCCTC-3′), and added ddH_2_O to 25 μL. PCR went for 27 cycles and the products were examined using agarose gel electrophoresis under 120 V for 20 min.

The SYBR^®^ Premix Ex TaqTM II (TliRNaseH Plus, Takara Bio, Mountain View, CA, USA) was used to RT-qPCR assays. The ABI PRISM 7500 was used to detect according to the manufacturer’s instruction (the program: 95 °C for 30 s; 95 °C for 5 s, 57 °C for 15 s, and 72 °C for 34 s, for 40 cycles; and a final extension at 72 °C for 10 min). The relative expression level was calculated as described by Livak and Schmittgen [47].

### 4.8. Western Blot Analysis on DmAMP1W Transgene Wheat

According to the plant protein extraction kit’s (CWBIO, Jiangsu, China) manufacturer’s instructions, total soluble proteins were extracted from the leaves of five transgenic and WT wheat lines. In brief, 200 μg of leaves each wheat line were crushed to fine powder in liquid nitrogen and resuspended in plant protein extraction reagent (990 μL) with a protease inhibitor cocktail (10 μL). Then, total soluble proteins were obtained by centrifugation (12,000 × *g*, 4 °C). Total soluble proteins (10 μL) from each line were heated for 10 min at 100 °C, and the proteins were separated on 15% SDS-PAGE and transferred to PVDF membrane (0.22 μm, Millipore, Boston, MA, USA).

The western blots were incubated with a 4000-fold dilution of Anti-His Mouse Monoclonal Antibody (TransGen Biotech, Beijing, China) at 4 °C and waved for 10 h. Subsequently, using the 4000-fold dilution of secondary antibody Goat Anti-Mouse IgG (H + L, TransGen Biotech, Beijing, China), conjugated to horseradish peroxidase at 25 °C and waved for 1 h. The DmAMP1W–His proteins were visualized using the ECL Western Blot Detection.

### 4.9. Wheat Sharp Eyespot and Common Root Rot Assessments

In T_1_-T_2_ generations, *DmAMP1W* transgenic wheat lines and recipient Zhoumai18 were inoculated with *R. cerealis* RC0301 as described by Chen et al. [3]. The infection types (ITs, it is graded from 0 to 5) and disease index (DI) were evaluated at the harvest stage as described by Zhu et al. [48].

Following the protocol of Dong et al. [49], wheat plant response assays with *B. sorokiniana* were carried out. In T_2_ generation, the experimental plants were planted in fields, and at the tillering stage, all the wheat plants were inoculated with *B. sorokiniana*. And the transgenic recipient Zhoumaii18 was used as control. Infection types (ITs, it is graded from 0 to 4) of wheat plants and disease index (DI) of a wheat line were evaluated at the harvest stage as described by Dong et al. [49].

## Figures and Tables

**Table 1:** Responses of *DmAMP1W* transgenic wheat lines to sharp eyespot.

Lines	T_1_		T_2_	
	IT	DI	IT	DI
DA1	2.17 **	43.41 **	2.50 **	50.00 **
DA2	1.81 **	36.27 **	2.57 **	51.40 **
DA3	2.22 *	44.36 *	2.72 *	54.40 *
DA4	1.72 **	34.47 **	2.30 **	46.00 **
DA5	1.37 *	27.31 *	2.68 *	53.60 *
WT	2.91	58.22	3.12	62.48

* or ** represent a significant difference between each transgenic wheat line and WT Zhoumai18 at *p* < 0.05 or 0.01 (Student’s *t*-test). IT represents infection type. DI represents eyespot disease index of each wheat line.

**Table 2:** Responses of *DmAMP1W* transgenic wheat to common root rot.

Lines	IT	DI
DA1	1.44 **	28.80 **
DA2	1.23 **	24.60 **
DA3	1.38 **	27.60 **
DA4	1.48 **	29.60 **
DA5	1.33 **	26.60 **
WT	2.27	45.40

** represents significant difference between each transgenic wheat line and WT Zhoumai18 at *p* < 0.01 (Student’s *t*-test). IT represents infection type. DI represents disease index of common root rot.

## Supplementary Materials

Supplementary Materials can be found at https://www.mdpi.com/1422-0067/21/2/647/s1.

## Abbreviations

AA	amino acid
AMP	antimicrobial peptide
DI	disease index
IPTG	isopropyl-*β*-D-thiogalactopyranoside
IT	infection type
MBP	maltose binding protein
RT-qPCR	reverse-transcription quantitative polymerase chain reaction
PBS	phosphate buffer saline
