# Supplementary material for: Ecotopic Expression of the Antimicrobial Peptide DmAMP1W Improves Resistance of Transgenic Wheat to Two Diseases: Sharp Eyespot and Common Root Rot
Source: Int J Mol Sci. 2020 Jan 18;21(2):647. doi: 10.3390/ijms21020647 (PMC7014311; doi:10.3390/ijms21020647)
Supplement: Supplementary file 1 [file ijms-21-00647-s001.pdf]

Ecotopic Expression of the Antimicrobial Peptide DmAMP1W Improves Resistance of Transgenic Wheat to two Diseases: Sharp Eyespot and Common Root Rot

Qiang Su, Ke Wang, Zengyan Zhang*

The National Key Facility for Crop Gene Resources and Genetic Improvement, Institute of Crop Sciences, Chinese Academy of Agricultural Sciences, Beijing 100081, China

Qiang Su: suqiangcaas@163.com

Ke Wang: wangke03@caas.cn

Zengyan Zhang: zhangzengyan@caas.cn

* **Correspondence:** Zengyan Zhang

Postal address: Institute of Crop Sciences, Chinese Academy of Agricultural Sciences, Zhongguancun South Street12, Beijing 100081, China

Supplementary data

Supplemental Figure S1 The coding sequence and amino acid sequence of DmAMP1W. Blue part represents the signal peptide (locating no. 1-28 AA residues). Green part represents the Knot1 domain (at 30 to 78 AA). Yellow part indicates (6×) His tag.

```
                            10        20        30        40        50        60
mRNA          1      ATGGTGAACAGGTCCGTGGCCTTCTCCGCCTTCGTGCTCATCCTCTTCGTGCTCGCCATC
Amino Acid    1       M  V  N  R  S  V  A  F  S  A  F  V  L  I  L  F  V  L  A  I

                            70        80        90       100       110       120
             61      TCCGACATCGCCTCCGTGTCCGGCGAGCTCTGCGAGAAGGCCTCCAAGACCTGGTCCGGC
             21       S  D  I  A  S  V  S  G  E  L  C  E  K  A  S  K  T  W  S  G

                           130       140       150       160       170       180
            121      AACTGCGGCAACACCGGCCACTGCGACAACCAATGCAAGTCCTGGGAGGGCGCCGCCCAC
             41       N  C  G  N  T  G  H  C  D  N  Q  C  K  S  W  E  G  A  A  H

                           190       200       210       220       230       240
            181      GGCGCCTGCCACGTGAGGAACGGCAAGCACATGTGCTTCTGCTACTTCAACTGCCATCAT
             61       G  A  C  H  V  R  N  G  K  H  M  C  F  C  Y  F  N  C  H  H

                           250
            241      CACCATCACCATTGA
             81       H  H  H  H  *
```

Supplemental Figure S2 Alignment of amino acid sequences of DmAMP1W and DmAMP1mature peptide (Accession: P0C8Y4). The software DANMAN was used to perform the sequence alignment.

```
DmAMP1_mature    ............................ELCEKASKTWSGNCGNT     17
DmAMP1W          MVNRSVAFSAFVLILFVLAISDIASVSGELCEKASKTWSGNCGNT     45
Consensus                                    elcekasktwsgncgnt

DmAMP1_mature    GHCDNQCKSWEGAAHGACHVRNGKHMCFCYFNC.....             50
DmAMP1W          GHCDNQCKSWEGAAHGACHVRNGKHMCFCYFNCHHHHH             83
Consensus        ghcdnqckswegaahgachvrngkhmcfcyfnc
```
